# Late onset depression: dopaminergic deficit and clinical features of prodromal Parkinson’s disease: a cross-sectional study

**DOI:** 10.1136/jnnp-2020-324266

**Published:** 2020-12-02

**Authors:** Hiba Kazmi, Zuzana Walker, Jan Booij, Faraan Khan, Sachit Shah, Carole H Sudre, Joshua E.J. Buckman, Anette-Eleonore Schrag

**Affiliations:** 1 Department of Clinical and Movement Neuroscience, UCL Institute of Neurology, London, UK; 2 Division of Psychiatry, University College London, London, UK; 3 St Margaret's Hospital, Essex Partnership University NHS Foundation Trust, Essex, UK; 4 Department of Radiology and Nuclear Medicine, Amsterdam UMC, Academic Medical Center, University of Amsterdam, Amsterdam, The Netherlands; 5 Atkinson Morley Regional Neuroscience Centre, St George's University Hospitals NHS Foundation Trust, London, UK; 6 Lysholm Department of Neuroradiology, National Hospital for Neurology and Neurosurgery, London, UK; 7 School of Biomedical Engineering and Imaging Sciences, King’s College London, London, UK; 8 Dementia Research Centre, Department of Neurodegenerative Disease, University College London Institute of Neurology, London, UK; 9 Centre for Outcomes Research and Effectiveness, Research Department of Clinical, Educational & Health Psychology, University College London, London, UK; 10 iCope, Camden and Islington Psychological Therapies Services, Camden & Islington NHS Foundation Trust, Camden & Islington NHS Foundation Trust, London, UK

## Abstract

**Background:**

Late onset depression (LOD) may precede the diagnosis of Parkinson’s disease (PD) or dementia with Lewy bodies (DLB). We aimed to determine the rate of clinical and imaging features associated with prodromal PD/DLB in patients with LOD.

**Methods:**

In a cross-sectional design, 36 patients with first onset of a depressive disorder (Diagnostic and Statistical Manual of Mental Disorders IV criteria) diagnosed after the age of 55 (LOD group) and 30 healthy controls (HC) underwent a detailed clinical assessment. In addition, 28/36 patients with LOD and 20/30 HC underwent a head MRI and 29/36 and 25/30, respectively, had dopamine transporter imaging by ^123^I-ioflupane single-photon emission computed tomography (SPECT) imaging. Image analysis of both scans was performed by a rater blind to the participant group. Results of clinical assessments and imaging results were compared between the two groups.

**Results:**

Patients with LOD (n=36) had significantly worse scores than HC (n=30) on the PD screening questionnaire (mean (SD) 1.8 (1.9) vs 0.8 (1.2); p=0.01), Movement Disorder Society Unified Parkinson’s Disease Rating Scale total (mean (SD) 19.2 (12.7) vs 6.1 (5.7); p<0.001), REM-sleep behaviour disorder screening questionnaire (mean (SD) 4.3 (3.2) vs 2.1 (2.1); p=0.001), Lille Apathy Rating Scale (mean (SD) −23.3 (9.6) vs −27.0 (4.7); p=0.04) and the Scales for Outcomes in PD-Autonomic (mean (SD) 14.9 (8.7) vs 7.7 (4.9); p<0.001). Twenty-four per cent of patients with LOD versus 4% HC had an abnormal ^123^I-ioflupane SPECT scan (p=0.04).

**Conclusions:**

LOD is associated with increased rates of motor and non-motor features of PD/DLB and of abnormal ^123^I-ioflupane SPECTs. These results suggest that patients with LOD should be considered at increased risk of PD/DLB.

## Introduction

Late onset depression (LOD) is defined as first onset of a depressive disorder after the age of 55 years with no history of significant depressive disorder. It has been postulated to be more likely due to an underlying organic brain disease than earlier onset depression.^1^ Vascular changes have been considered in the pathology of LOD with reports of increased white matter lesions in patients with LOD,^2 3^ although this is not universally accepted.^4^ Studies have also suggested that up to 40% of patients with LOD develop dementia, including Alzheimer’s disease (AD) and dementia with Lewy bodies (DLB).^5^

At least 10 years prior to the emergence of motor features, non-motor symptoms can present during the prodromal phase of Parkinson’s disease (PD), such as autonomic dysfunction, sleep disorder (REM-sleep behaviour disorder (RBD)), loss of sense of smell, and neuropsychiatric features including anxiety, depression and apathy.^6^ Retrospective studies have reported depression and anxiety up to two decades^7^ prior to PD diagnosis, and in register-based studies diagnosis of depressive disorders has been associated with a significantly higher rate of PD,^8^ suggesting depressive disorders starting in later life can represent the first presentation of PD. However, no study to date has investigated whether clinical or imaging markers suggestive of prodromal PD or DLB are increased specifically in LOD. Using clinical assessments and dopamine transporter imaging, we hypothesised that patients with LOD would have an increased rate of clinical features and dopaminergic deficit typically associated with PD or DLB.

## Methods

### Patients

Between March 2015 and June 2017, 36 patients aged 55 years and older were recruited from old-age psychiatry services, primary care services, voluntary organisations and via public advertising in the UK. Inclusion criteria were a current or previous clinical diagnosis of either major depressive disorder, mixed anxiety and depressive disorder, minor depressive disorder or dysthymia (as per the Diagnostic and Statistical Manual of Mental Disorders IV criteria), with onset after the age of 55 years and a disease duration of ≤10 years. Exclusion criteria were an existing diagnosis of PD or a neurodegenerative condition, a mood disorder as a reaction to a severe life event such as a bereavement or a life-threatening illness, a diagnosis of bipolar affective disorder and/or medication-induced depression or untreated systemic disorders (eg, hypothyroidism). Thirty healthy controls (HC) without evidence of a depressive disorder and without a diagnosis of a neurodegenerative condition were also recruited. Standard exclusion criteria for imaging studies were applied.

### Standard protocol approvals, registrations and patient consents

Permission to use radioactive substances was obtained from the Radioactive Substances Advisory Committee (ARSAC).

### Role of the funding source

The funders had no involvement in the study design, in the collection, analysis and interpretation of data or in the writing of the report. The corresponding author has full access to all the data in the study and had final responsibility for the decision to submit for publication.

### Clinical assessment

Participants were assessed using the following: Patient Health Questionnaire 9 (PHQ-9), Hospital Anxiety and Depression Scale (HADS), Movement Disorder Society Unified Parkinson’s Disease Rating Scale (MDS UPDRS) (conducted by a researcher experienced and trained in undertaking the MDS-UPDRS assessments) Montreal Cognitive Assessment (MoCA), Frontal Assessment Battery (FAB), Lille Apathy Rating Scale (LARS), REM Sleep Behaviour Disorder Single-Question Screen (RBD1Q), The REM Sleep Behaviour Disorder Screening Questionnaire (RBDSQ), Parkinson’s Disease Sleep Scale (PDSS), Parkinson’s disease screening questionnaire (PD screening), Scales for Outcomes in Parkinson’s disease-Autonomic (SCOPA-AUT) and the University of Pennsylvania Smell Identification Test (UPSIT), UK version (for references, see online supplemental material). Demographic data included family history of PD or dementia, a history of smoking, cardiovascular risk score (QRISK2-2017; https://qrisk.org/2017/) and medication history.

10.1136/jnnp-2020-324266.supp1 Supplementary data

The FAB was not completed by three patients with LOD and the PDSS by one HC. One HC was excluded from the MDS UPDRS motor examination subscale as they could not complete the full examination due to a leg injury.

The following cut-offs were used to denote abnormal scores on clinical assessments: PHQ-9 ≥5, MoCA <23, FAB <12, LARS ≥−21, HADS (depression (HADS-D) and anxiety (HADS-A) rated separately) each ≥8, RBDSQ≥5, the single RBD questionnaire (RBD1Q; a score of “yes” on the following question; “Have you ever been told, or suspected yourself, that you seem to "act out your dreams” while asleep (for example, punching, flailing your arms in the air, making running movements, etc.)?”. Items from the MDS-UPDRS motor examination were used to calculate subthreshold parkinsonism/mild parkinsonian signs (MPS)≥6, and a cut-off of ≥5 was used for the PD screening questionnaire. For the UPSIT, participants with a score ≤the 15th percentile of an age and gender matched population were classed as having hyposmia. Cut-off scores for the PDSS (<99.7) and the SCOPA-AUT (>14.2) were calculated scores±1 SD from control group means in the original validation studies (PDSS; 120.7 (SD 21), SCOPA-AUT; 8.8 (SD 5.4). In addition to using the MDS UPDRS motor examination items to calculate MPS, subscores were calculated for axial symptoms, bradykinesia, rigidity and tremor (for references, see online supplemental material).

### ^123^I-ioflupane SPECT protocol

**Figure 1:**
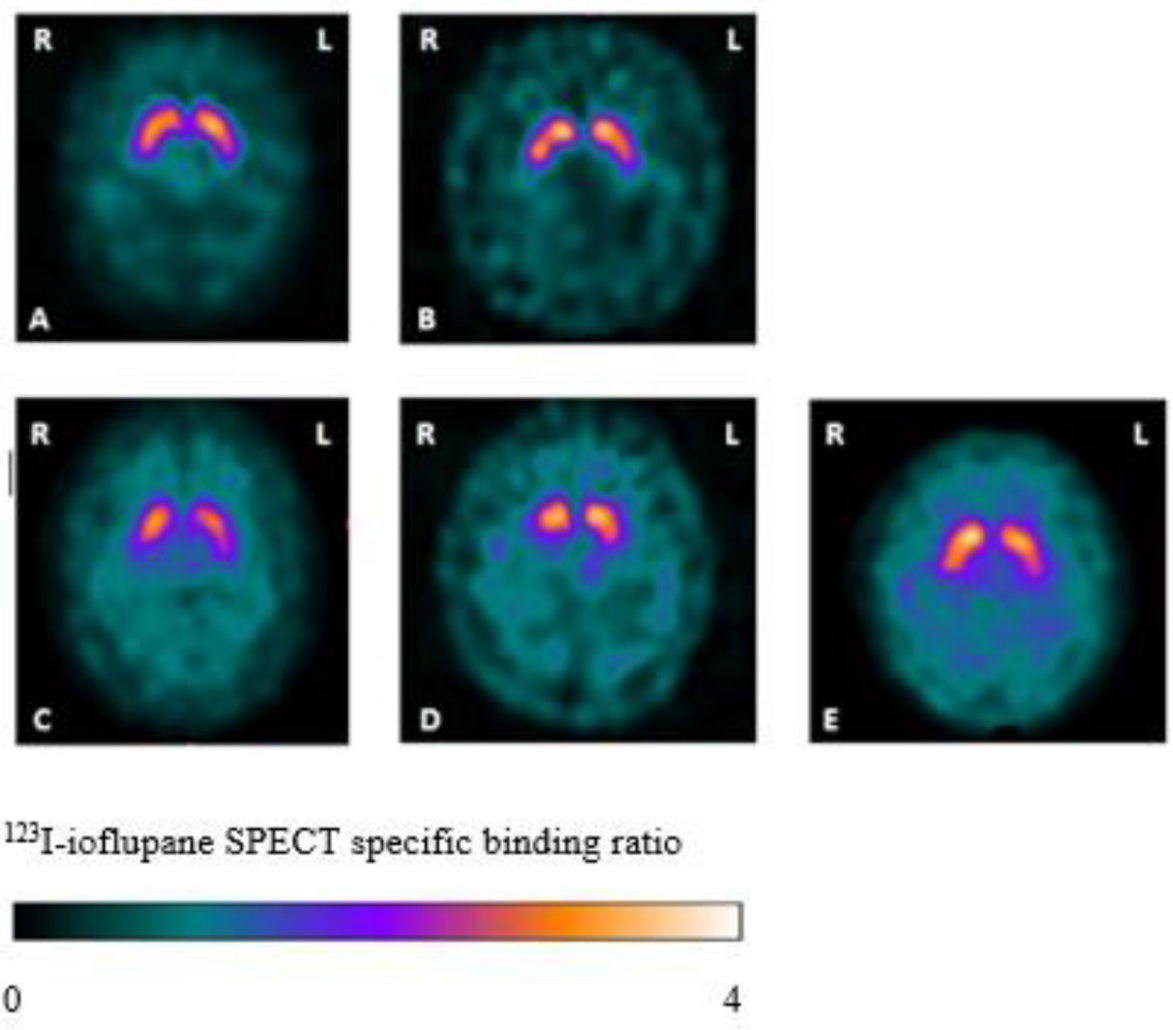
Examples of ^123^I-ioflupane SPECTs from the study and their visual ratings (transversal SPECT slices at the level of the striatum). (A) Normal. (B) Equivocal. (C) Abnormal type 1. (D) Abnormal type 2. (E) Balanced striatal loss. L, left side; R, right side.

Twenty-nine patients with LOD and 25 HC underwent dopamine transporter imaging with ^123^I-ioflupane SPECT, performed at two different research sites, both using the Siemens Symbia Dual Headed Scanner (with a high-resolution parallel hole collimator). Prior to the scan, participants were given thyroid blocking medication. A single intravenous ^123^I-ioflupane SPECT injection (DaTSCAN, GE Healthcare, Amersham UK) was administered with a volume of 2.5 mL and radioactivity between 111–185 MBq. Three to 4 hours after the injection, participants underwent a SPECT scan measuring binding to the presynaptic dopamine transporter in the striatum. SPECT was acquired in a 128*128 matrix obtaining 120 frames over 360**°**. The scans were reconstructed using GE Healthcare DaTQUANT with OSEM 2×10 with a Butterworth filter 0.6 cut-off and corrected for attenuation by the Chang method. Blinded visual assessment of the images was performed according to published criteria^9^ (figure 1) with images categorised as normal (including equivocal; striatal dopamine transporter binding slightly reduced but within normality and possibly due to a normal variant or a consequence of age), abnormal type 1 (asymmetric activity, eg, activity in the region of the putamen of one hemisphere is absent or greatly reduced with respect to the other), abnormal type 2 (activity is absent in the putamen of both hemispheres and confined to the caudate nuclei), abnormal type 3 (activity absent in the putamen of both hemispheres and greatly reduced in one or both caudate nuclei). An additional rating of balanced striatal loss (balanced loss to the caudate nuclei and putamen with high levels of back ground activity) was included, given reports of this pattern of uptake present in those with DLB.^10^

### MRI

Twenty-eight patients with LOD and 20 HC also had a structural MRI head scan using either a Philips 1.5 T (n=36) or 3 T (n=12) scanner at two sites using the following sequences; 3D T1 (175 slices, thickness 1 mm, Field of view 240×240×175), T2, FLAIR and T2FFE (22 slices, thickness 6 mm, FOV 230×184×145). Blinded visual ratings were undertaken of the MR images for white matter lesions and atrophy using Fazekas, Koedam and global cortical atrophy (GCA) and medial temporal atrophy (MTA) rating scales.^11–14^ Additionally, the Geodesic Information Flows framework^15^ for brain parcellation was applied to calculate subcortical volumes for the following regions: third and fourth ventricles and left and right volumes of the putamen, caudate amygdala and hippocampus. Hippocampus volumes included the dentate gyrus, the ammonic subfields (CA1, CA2, CA3, and CA4), the prosubiculum and the subiculum. Global white matter and grey matter volumes were also calculated by the sum of the white and grey matter regions, respectively. The volumes were provided in cubic millimetres (mm³). White matter lesion load was also investigated by coregistering T1 and Fluid-attenuated inversion recovery (FLAIR) sequences for optimal segmentation and then obtained using the using the Bayesian Model Selection (BaMoS) framework.^16^

### Statistical analysis

The main analysis was the comparison between patients with LOD and HCs. Histograms and QQ plots were used to check the distribution of variables. For continuous data, independent sample t-tests for normally distributed data and Mann-Whitney U tests for non-normally distributed data were used. χ^2^ tests and Fisher’s exact tests (for smaller numbers) were used for comparison of categorical data. To account for the effect of medications on motor, RBD and hypotension/autonomic dysfunction related clinical scores, secondary analysis was run with participants on the relevant medications excluded. For the volumetric MR analysis, subcortical volumes and white matter lesion load were log transformed and differences in white matter lesion load, subcortical and global WM and GM volumes between groups were analysed using analysis of covariance (ANCOVA). The following variables were included as covariates: age, gender, total intracranial variability and the type of scanner used (as one site used a 1.5 T and another site a 3T scanner). Analyses were performed using SPSS V.22.

## Results

The demographic details and cardiovascular risk factors of the two groups are listed in table 1. All were comparable, although those in the LOD group took more antihypertensive medication. Ten patients with LOD had a family history of dementia (eight first degree relatives) and two had relatives with PD (one with a first degree relative). Four HC had a family history of dementia (two with a first degree relative) and two HC had a second degree relative with PD.

**Table 1:** Baseline characteristics

	LOD (n=36)	HC (n=30)	P value
Mean (SD) age in years	67.4 (7.8)	70.6 (9.3)	0.13
Male/female n (%)	14 (39)/22 (61)	11 (37)/19 (63)	0.53
Caucasian n (%)	32 (89)	29 (97)	0.45
Family history of PD/dementia (%)	12 (33)	6 (20)	0.18
<12 years education n (%)	10 (28)	7 (23)	0.45
Mean Q risk score† (SD)	18.6 (11.0)	19.3 (11.6)	0.92
Antihypertensive treatment n (%)	15 (42)	6 (20)	0.05
Diabetes mellitus n (%)	5 (13.9)	3 (10)	0.46
Current/previous smoker* n (%)	20 (55)	12 (40)	0.16

Independent samples t-test, Mann-Whitney U test and Fisher’s exact

*Current and ex-smoker versus non-smoker.

†Cardiovascular risktest calculator using age, sex, ethnicity, smoking status, diabetes statusand evidence of; chronic kidney disease, atrial fibrillation, blood pressuretreatment and Rheumatoid arthritis.

LOD, patients with late onset depressive disorder; HC, healthy controls; PD, Parkinson’s disease

Patients with LOD had been experiencing symptoms of depression/anxiety for a mean (SD) of 3.3 years (±2.8) with 30 (83%) having active depression as assessed by the PHQ-9. However, on average the symptoms were mild (mean (SD) 9.1 (6.3)). Seventeen (47%) of patients with LOD were taking medication(s) for the treatment of symptoms associated with depression/anxiety; 13 (36%) were taking a selective serotonin reuptake inhibitor; three (8%) a serotonin and norepinephrine reuptake inhibitor; 2 (6%) a tricyclic agent; 2 (6%) a neuroleptic, and 1 (3%) an anticonvulsant as a mood stabiliser.

### Motor symptoms

The LOD group had significantly higher scores (mean (SD)) than the HC group on the PD screening scale (1.8 (1.9) vs 0.8 (1.2); p=0.01) and the total MDS-UPDRS (19.2 (12.7) vs 6.1 (5.7); p<0.001) (table 2). On average, the LOD group also scored significantly higher on all the MDS-UPDRS subscales, including part I: nM-Experiences of Daily Living (EDL; mean 14.0 (SD 8.6) vs 4.1 (3.5); p<0.001), part II: M-EDL (3.5 (3.9) vs 1.3 (2.0); p=0.007), and the motor examination score (2.3 (2.7) vs 1.3 (2.0); p=0.003) (part III). Group differences on the MPS score did not reach significance. There were also significant differences in part III subscores for rigidity (0.7 (1.2) vs 0.1 (0.4); p=0.005) and bradykinesia (1.2 (1.8) vs 0.5 (1.1); p=0.02).

**Table 2:** Assessment scores of motor features in patients with late onset depressive disorder (LOD) and healthy controls (HC)

Assessment	LOD (n=36)	HC (n=30)	P value
	Mean (SD)		
PD screening questionnaire	1.8 (1.9)	0.8 (1.2)	0.01
MDS-UPDRS Total	19.2 (12.7)	6.1 (5.7)	<0.001
Non motor-EDL (part I)	14.0 (8.6)	4.1 (3.5)	<0.001
Motor-EDL (part II)	3.5 (3.9)	1.3 (2.0)	0.007
Motor examination (part III)	2.3 (2.7)	0.7 (1.3)*	0.003
Sum of axial subscore*	0.19 (0.5)	0.03 (0.2)	0.15
Sum of bradykinesia subscore*	1.23 (1.8)	0.52 (1.1)	0.02
Sum of rigidity subscore	0.67 (1.2)	0.10 (0.4)	0.005
Sum of tremor subscore	0.14 (0.4)	0.07 (0.4)	0.26

Mann-Whitney U test.

*One HC did not complete the MDS-UPDRS motor examination.

EDL, Experiences of Daily Living; MDS-UPDRS, Movement Disorder Society Unified Parkinson’s Disease Rating Scale; PD, Parkinson’s disease.

### Non-motor symptoms

As expected, patients with LOD had significantly higher scores (mean (SD)) than HC on the HADS depression (7.1 (4.6) vs 1.2 (1.0); p<0.001) and HADS anxiety scales (9.6 (5.1) vs 1.2 (1.0); p<0.001) and had higher PHQ-9 scores (9.1 (6.3) vs 1.4 (1.9); p=0.02) (table 3). The LOD group also had significantly higher scores than HC on the RBDSQ (4.3 (3.2) vs 2.1 (2.1); p=0.001), and the RBD1Q (10 (27.8%) vs 2 (6.7%); p=0.02). The LOD group had worse overall sleep dysfunction as assessed on the PDSS (98.1 (SD 24.4) vs 127.6 (13.1); p<0.001), and apathy scores on the LARS than HC (mean −23.3 (SD 9.6) vs -27.0 (4.7); p=0.04). On average patients with LOD also had worse autonomic dysfunction than HC (SCOPA AUT; 14.9 (8.7) vs 7.7 (4.9); p<0.001). There were no significant group differences on the MoCA, FAB or UPSIT.

**Table 3:** Assessment scores of non-motor symptoms in patients with late onset depressive disorder (LOD) and healthy controls (HC)

Assessment	LOD (n=36)*	HC (n=30)*	P value
RBDSQ mean (SD)	4.3 (3.2)	2.1 (2.1)	0.001
RBD1Q “yes” n (%)	10 (27.8)	2 (6.7)	0.02
SCOPA-AUT mean (SD)	14.9 (8.7)	7.7 (4.9)	<0.001
PDSS mean (SD)	98.1 (24.4)	127.6 (13.1)	<0.001
UPSIT mean (SD)	30.7 (5.7)	29.5 (6.6)	0.43
MoCA mean (SD)	26.0 (3.6)	26.5 (2.7)	0.51
FAB mean (SD)	17.1 (1.1)	17.0 (1.3)	0.85
LARS mean (SD)	−23.3 (9.6)	−27.0 (4.7)	0.04
PHQ-9 mean (SD)	9.1 (6.3)	1.4 (1.9)	0.02
HADS-D mean (SD)	7.1 (4.6)	1.2 (1.0)	<0.001
HADS-A mean (SD)	9.6 (5.1)	2.8 (2.8)	<0.001

Independent samples t test, Mann-Whitney U test and Fisher’s exact.

*Three LOD did not complete the Frontal Assessment Battery (FAB) and one HC did not complete the PDSS.

HADS, Hospital Anxiety and Depression Scale; HADS-A, HADS anxiety subscale; HADS-D, HADS depression subscale; LARS, Lille Apathy Rating Scale; MoCA, Montreal Cognitive Assessment; PDSS, Parkinson’s Disease Sleep Scale; PHQ-9, Patient Health Questionnaire-9; RBD1Q, REM Sleep Behaviour Disorder Single-Question Screen; RBDSQ, The REM Sleep Behaviour Disorder Screening Questionnaire; SCOPA-AUT, Scales for Outcomes in Parkinson’s Disease-Autonomic; UPSIT, University of Pennsylvania Smell Identification Test.

After excluding participants on medications that could contribute to motor and non-motor features such as RBD or hypotension/autonomic dysfunction scores, the group differences in the clinical scores remained significant in the UPDRS motor examination (p=0.004), the RBDSQ (p=0.02) and the SCOPA-AUT (p=0.001) (online supplemental data; online supplemental table e1).

Online supplemental figure e1 (online supplemental data) shows the number of participants that had abnormal scores on the clinical scales when using cut-offs. An analysis dichotomising the clinical scores showed similar results.

### ^123^I-ioflupane SPECT imaging

The 29 patients with LOD and 25 HC who had a ^123^I-ioflupane SPECT had comparable demographics and clinical scores to the overall group (online supplemental data; online supplemental table e2).

There was a significantly higher number of patients with LOD who had ^123^I-ioflupane SPECT with abnormal visual ratings than HC (p=0.04; table 4). These included two patients with LOD with abnormal type 1, two with type 2 and three with balanced striatal loss. One HC had an abnormal ^123^I-ioflupane SPECT with balanced striatal loss. Twenty-two patients with LOD were rated as having normal scans including two rated as equivocal. Twenty-four HCs were rated as having normal scans including three rated as equivocal.

**Table 4:** ^123^I-ioflupane SPECT visual ratings in patients with late onset depressive disorder (LOD) and healthy controls (HC)

^123^I-ioflupane rating	Rating type	LOD (n=29) n (%)	HC (n=25) n (%)	P value
Normal	Normal	20 (69)	21 (84)	
	Equivocal	2 (7)	3 (12)	
	Total normal	22 (76)	23 (96)	
Abnormal	Abnormal type 1	2 (7)	0 (0)	
	Abnormal type 2	2 (7)	0 (0)	
	Abnormal type 3	0 (0)	0 (0)	
	Striatal loss	3 (10)	1 (4)	
	Total abnormal	7 (24)	1 (4)	0.04

Fisher’s exact comparing number of normal and abnormal ^123^I-ioflupane SPECTs.

In the LOD group, those with abnormal ^123^I-ioflupane SPECT had significantly worse scores than those with normal ^123^I-ioflupane SPECT on the UPDRS motor part III, MoCA and FAB assessments and UPSIT scores (all p<0.05) (tables 5–7).

**Table 5:** Motor features scores in patients with late onset depressive disorder (LOD) with abnormal versus normal ^123^I-ioflupane SPECT ratings

Clinical assessments	Abnormal (n=7)	Normal (n=22)	P value
	Mean (SD)		
PD screening questionnaire	2.9 (2.7)	1.7 (1.7)	0.41
MDS UPDRS total	22.1 (15.0)	19.8 (12.9)	0.71
Non-motor EDL (part I)	13.4 (10.6)	14.1 (8.3)	0.77
Motor-EDL (part II)	4.1 (3.8)	3.4 (4.3)	0.41
UPDRS motor examination (part III)	4.6 (2.2)	2.2 (2.7)	0.01
Sum of axial subscore	0.43 (0.79)	0.18 (0.50)	0.57
Sum of bradykinesia subscore	1.71 (0.49)	1.5 (2.22)	0.14
Sum of rigidity subscore	2.29 (1.70)	0.27 (0.55)	0.002
Sum of tremor subscore	0.14 (0.38)	0.18 (0.50)	1.00

Mann-Whitney U test.

EDL, Experiences of Daily Living; LOD, late onset depressive disorder; MDS-UPDRS, Movement Disorder Society Unified Parkinson’s Disease Rating Scale; PD, Parkinson’s disease.;

**Table 6:** Non-motor symptoms scores in patients with late onset depressive disorder (LOD) with abnormal versus normal ^123^I-ioflupane SPECT ratings

Clinical assessments	Abnormal (n=7)	Normal (n=22)	P value
RBDSQ mean (SD)	4.4 (3.3)	4.9 (3.4)	0.86
RBD1Q n (%)	3 (42.8)	7 (31.8)	0.46
PDSS mean (SD)	102.7 (20.6)	93.0 (26.2)	0.38
UPSIT mean (SD)	21.7 (4.9)	33.3 (3.2)	<0.001
MoCA mean (SD)	22.1 (3.5)	26.2 (3.1)	0.01
FAB mean (SD)	16.2 (1.9)*	17.4 (0.7)*	0.02
LARS mean (SD)	−19.0 (11.9)	−23.2 (9.7)	0.26
PHQ-9 mean (SD)	9.1 (7.9)	9.5 (6.4)	0.78
HADS-D mean (SD)	9.3 (6.1)	6.7 (4.5)	0.44
HADS-A mean (SD)	8.9 (5.1)	10.3 (5.5)	0.60
SCOPA-AUT mean (SD)	19.3 (11.2)	13.0 (7.3)	0.20

Independent samples t-test, Mann-Whitney U test and Fisher’s exact test.

*Two abnormal ^123^I-ioflupane SPECT patients with LOD and one normal ^123^I-ioflupane SPECT patient with LOD did not complete the Frontal Assessment Battery (FAB).

HADS, Hospital Anxiety and Depression Scale; HADS-A, HADS anxiety subscale; HADS-D, HADS depression subscale; LARS, Lille Apathy Rating Scale; MoCA, Montreal Cognitive Assessment; PDSS, Parkinson’s Disease Sleep Scale; PHQ-9, Patient Health Questionnaire-9; RBD1Q, REM Sleep Behaviour Disorder Single Questionnaire; RBDSQ, The REM sleep Behaviour Disorder Screening Questionnaire; SCOPA-AUT, Scales for Outcomes in Parkinson’s Disease-Autonomic; UPSIT, University of Pennsylvania Smell Identification Test.

**Table 7:** Abnormal clinical scores in participants with an abnormal ^123^I-ioflupane SPECT (late onset depression (LOD) n=7, healthy controls (HC) n=1)

Participant group	Age in years	Male/ female	Family history	PD screening questionnaire	MPS	RBDSQ	RBD1Q	SCOPA-AUT	MoCA	FAB	UPSIT percentile	LARS	PDSS	HADS-A	HADS-D	PHQ-9	LOD duration (years)
LOD	60	M		A				A			13th		A	A	A	A	2
LOD	63	M		A		A	A		22	N/A	11th	A	A	A	A	A	4
LOD	64	M	D				A				15th	A	A	A		A	10
LOD	76	M		A				A	19	N/A		A			A		2
LOD	76	M							16		15th						6
LOD	77	M			A	A	A	A			10th			A			2
LOD	79	M			A							A		A	A	A	2
HC	56	M	PD														–

A, abnormal score; D, dementia; FAB, Frontal Assessment Battery; HADS, Hospital Anxiety and Depression Scale; HADS-A, HADS Anxiety Subscale; HADS-D, HADS Depression Subscale; LARS, Lille Apathy Rating Scale; MoCA, Montreal Cognitive Assessment; MPS, Mild Parkinsonian Signs; N/A, missing data; PD, Parkinson’s disease; PDSS, Parkinson’s Disease Sleep Scale; PHQ-9, Patient Health Questionnaire-9; RBD1Q, REM Sleep Behaviour Disorder Single-Question Screen; RBDSQ, The REM sleep Behaviour Disorder Screening Questionnaire; SCOPA-AUT, Scales for Outcomes in Parkinson’s disease-Autonomic; UPSIT, University of Pennsylvania Smell Identification Test.

### Structural MR imaging

There were no significant differences between patients with LOD (n=28) and HC (n=20) in mean ratings of atrophy (GCA, Koedam and MTA scales) or white matter lesions (Fazekas scale). Volumetric measurement of the right amygdala and hippocampus showed a trend for smaller volumes in the LOD group, but there were no differences in volumes of the third and fourth ventricles, putamen, caudate nucleus, global white and grey matter and white matter lesion load (online supplemental data; online supplemental table e3).

### One year clinical follow up

All participants were invited for review at 1 year. However, only 13 patients with LOD and 9 HC participated. None reported a diagnosis of PD or DLB or other neurodegenerative disorder. Change in clinical assessment scores from baseline to the 1-year follow-up was expressed as differences of mean scores for both the LOD and HC groups. There were significant group differences between the change in HADS-A and HADS-D scores, which increased in HC and decreased in patients with LOD. On the other hand, RBDSQ and SCOPA-AUT scores significantly increased in the LOD group and decreased in the HC. There was also a greater increase in the MDS-UPDRS scores in the LOD group (mean change 3.2 (SD 8.1) than in HC (mean change 0.8 (SD 0.5)) but this difference was not statistically significant.

## Discussion

This study shows that LOD is associated with increased rates of both motor and non-motor prodromal features of PD and DLB and with abnormal ^123^I-ioflupane SPECT scan findings typically seen in PD and DLB. While the reported symptoms of anxiety, apathy and sleep dysfunction may be explained by the depressive disorder alone, cognitive impairment and symptoms of autonomic dysfunction including constipation and urinary symptoms are unlikely to be seen in depressive disorders other than in severe forms and our cohort had mild symptoms. This makes it unlikely that most of the non-motor features were due to a depressive syndrome alone. However, they are recognised features of the prodromal phase of PD and DLB. The LOD group also had significantly higher rates of more specific features of prodromal PD and DLB, including RBD (RBDSQ and RBD1Q) and motor features. Rigidity and bradykinesia scores, and the self-completed parts of MDS-UPDRS showed significantly higher scores in LOD group than in HC. It is noteworthy that differences were not significant in the axial features with general body bradykinesia (slowing of speech, loss of facial expression and body bradykinesia), as typically seen in patients with depression, but were particularly present in the rigidity and limb bradykinesia scores. In addition, patients with LOD also scored significantly higher on the specific PD-screening questionnaire.

A blinded evaluation of ^123^I-ioflupane SPECT demonstrated an underlying nigrostriatal deficit in 7 out of 29 patients with LOD with patterns usually associated with PD^9^ and DLB.^10^ Previous investigations of striatal dopamine transporter in those with major depression (with no studies specific to LOD) have reported mixed results, possibly due to inclusion of different populations of patients, types of depressive disorder and the use of different imaging tracers. The majority of studies using ^99m^Tc-TRODAT-1 reported greater dopamine transporter availability in the striatum in patients with major depressive disorder and in patients with bipolar affective disorder.^17–19^ However, studies using the ^123^I-β-CIT SPECT radioligand in patients with depression and controls have reported conflicting results with reports of a reduction in dopamine transporter binding in the left striatum in patients with symptoms of depression with a seasonal affective disorder^20^ but also significantly higher dopamine transporter binding on both sides of the striatum in 15 drug free patients with major depression with young age of onset in comparison to controls.^21^ To our knowledge, only one small study has previously performed ^123^I-ioflupane SPECT imaging in patients with depressive disorder.^22^ Dopamine transporter binding in 11 patients with a major depressive disorder who had anhedonia as a predominant feature (mean age of 41.1, SD 11.9; with patients over 60 years of age excluded) compared with nine HC, reported that dopamine transporter binding in the striatum was reduced bilaterally.^22^

The association between depression and the subsequent risk of developing PD is well described.^8 23^ The possibility that LOD is a prodromal feature of PD or DLB is further strengthened by this study which shows that the clinical features of motor and non-motor dysfunction seen in PD were more commonly present in those patients with LOD with an abnormal dopamine transporter scan than in those with a normal scan. All our patients with LOD with abnormal ^123^I-ioflupane SPECT findings had clinical features previously described in prodromal PD and DLB,^24–26^ suggesting that they reflect the underlying disease process in these patients.

Using MRI, a number of studies have reported loss of hippocampal volumes in major depression compared with HCs,^27 28^ particularly in those with LOD,^28–30^ suggesting an association with AD. An association between LOD and AD has further been supported by a large epidemiological study that reported that 41% of older patients with MCI and recent active depression progressed to AD in comparison to 32% of patients with a remote history of depression.^31^ A further study reported that higher plasma Aβ42 at baseline was a positive predictor for the first episode of LOD in patients with mean age of 75.8 years (SD 0.5).^32^ In our study, there was a trend for reduced right amygdala and hippocampal volumes in the LOD group which did not reach significance. Increased vascular pathology with increased white matter lesions^33–35^ has also been reported in LOD. In contrast, in this study, we did not find any difference in vascular pathology or risk factors associated with LOD. These findings are in keeping with autopsy studies that found no evidence of cerebrovascular or AD-related lesions in patients with late life depression.^36^ However, our study was not specifically designed to address these questions and more specific studies with larger sample sizes and dedicated imaging modalities are required to investigate the association of LOD and other vascular and neurodegenerative disorders.

### Limitations

The main limitation of this study is its cross-sectional nature. However, given the slowly progressive course of early PD, only a very large or long-term study would allow definite validation that LOD with abnormal ^123^I-ioflupane SPECT is a precursor of PD or DLB. Another limitation was the use of the RBD questionnaires. Only a polysomnography can definitely confirm clinical RBD. It is however unlikely that there was a bias towards greater rates of RBD using both the full RBDSQ and the short RBD1Q.

Use of different MR scanners (1.5 T and 3 T) may have reduced the likelihood of finding abnormalities. In addition, it is possible that medications may have affected some clinical and ^123^I-ioflupane SPECT findings. However, group differences persisted when those taking medications potentially affecting clinical ratings were excluded, and antidepressants and neuroleptics are not thought to significantly interfere with visual ratings of ^123^I-ioflupane SPECT.^37 38^ Finally, it should be noted that the sample size used in this pilot study was small and confirmation of the results with a bigger sample size is recommended.

In conclusion, our results suggest that individuals with LOD should be considered at increased risk of developing PD or DLB. The approach for these patients should be similar to that for patients with RBD or anosmia.
