# Supplementary material for: Late onset depression: dopaminergic deficit and clinical features of prodromal Parkinson’s disease: a cross-sectional study
Source: J Neurol Neurosurg Psychiatry. 2020 Dec 2;92(2):158–64. doi: 10.1136/jnnp-2020-324266 (PMC7841491; doi:10.1136/jnnp-2020-324266)
Supplement: Supplementary data [file jnnp-2020-324266supp001.pdf]

Table e-1 Mean scores clinical measures when excluding patients with late onset depressive disorder (LOD) and healthy controls (HC) on medications potentially affecting these scores*

Clinical assessment	LOD	HC	*P*
	**n=33**	**n=24**	
**UPDRS Motor Examination mean (SD)**	0.7 (1.5)	1.9 (2.5)	.004
	**n= 19**	**n=30**	
**RBD1Q "yes" n (%)**	5 (13.8)	2 (6.6)	.08
**RBDSQ mean (SD)**	4.1 (3.1)	2.1 (2.1)	.02
	**n=26**	**n=24**	
**SCOPA-AUT mean (SD)**	14.3 (7.7)	7.6 (5.1)	.001

Mann Whitney U test, Fisher's Exact test
Abbreviations UPDRS= Unified Parkinson's Disease Rating scale, RBD1Q= REM Sleep Behavior Disorder Single-Question Screen, RBDSQ= The REM sleep Behaviour Disorder screening questionnaire, SCOPA-AUT= Scales for Outcomes in Parkinson's disease- Autonomic.
*Potentially affecting RBDSQ, RBD1Q; Citalopram, Sertraline, Escitalopram, Venlafaxine, Fluoxetine, Nortriptyline, Amytriptyline, Paroxetine, Olanzapine and Risperidone, SCOPA AUT: Nortriptyline, Risperidone, Olanzapine, Pregabalin, Lisinopril, Atenolol, Losartan, Amlodipine, Ramipril, Bisoprolol and Bendroflumethiazide, MDS-UPDRS motor examination: Olanzapine, Risperidone and Amlodipine.

Table e-2 Baseline characteristics of patients with late onset depressive disorder (LOD) and healthy controls (HC) who had a ^{123}I-ioflupane SPECT

	LOD (n=29)	HC (n=25)	*p*
**Age in years mean (SD)**	67.7 (7.5)	69.7 (9.0)	.37
**Male/ Female n (%)**	13 (45)	9 (36)	.35
**Caucasian n (%)**	25 (86.2)	24 (96)	.23
**Family history of PD/ dementia n (%)**	9 (31)	6 (24)	.40
**<12 years education n (%)**	8 (27.6)	5 (20)	.37
**Q risk score ^ mean (SD)**	18.8 (11.6)	19.2 (12.1)	.96
**Anti-hypertensive treatment n (%)**	10 (34.5)	4 (16.4)	.11
**Diabetes n (%)**	5 (17)	3 (12)	.44
**Current/ Previous smoker n (%)**	15 (51.7)	10 (40)	.28*

Independent samples t test, Mann Whitney U test and Fisher's exact
^Q risk score=Cardiovascular risk test calculator (https://qrisk.org/2017/, 2017), using the age, sex, ethnicity, smoking status, diabetes status and evidence of; chronic kidney disease, atrial fibrillation, blood pressure treatment and Rheumatoid arthritis, 2 HC did not complete the q risk
*Current and ex-smoker vs Non smoker
Abbreviations; PD =Parkinson's disease

Table e-3 Comparison of MRI findings between patients with late onset depressive disorder (LOD) and healthy controls (HC)

Region	LOD	HC	*p*
	**Mean mm³ (SD)<br>Median [1st IQ; 3rd IQ])**		
**Third Ventricle**	3.11 (0.15)	3.15 (0.12)	.20
	3.11 [3.01;3.22]	3.18 [3.04; 3.25]	
**Fourth Ventricle**	3.27 (0.11)	3.27 (0.10)	.48
	3.27 [2.20;3.33]	3.26 [3.19;3.36]	
**Right Caudate**	3.49 (0.05)	3.49 (0.05)	.47
	3.48 [3.46;3.53]	3.48 [3.43;3.51]	
**Left Caudate**	3.46 (0.05)	3.47 (0.05)	.89
	3.45 [3.41;3.49]	3.46 [3.43;3.51]	
**Right Putamen**	3.59 (0.04)	3.59 (0.02)	.19
	3.59 [3.55;3.63]	3.59 [3.57;3.62]	
**Left Putamen**	3.60 (0.05)	3.60 (0.03)	.26
	3.60 [3.56;3.63]	3.60 [3.58;3.63]	
**Right Hippocampus**	3.55 (0.04)	3.58 (0.03)	.08
	3.56 [3.52;3.58]	3.57 [3.54;3.61]	
**Left Hippocampus**	3.54 (0.05)	3.55 (0.03)	.45
	3.54 [3.50;3.59]	3.55 [3.53;3.59]	
**Right Amygdala**	3.18 (0.05)	3.21 (0.03)	.07
	3.20 [316;3.21]	3.21 [3.2;3.24]	
**Left Amygdala**	3.19 (0.05)	3.21 (0.03)	.24
	3.19 [3.17;3.24]	3.21 [3.18;3.24)	
**Total white matter**	5.61 (0.05)	5.61 (0.05)	.30
	5.61 [5.58;5.65]	5.61 [5.57;5.65]	
**Total grey matter**	5.64 (0.03)	5.65 (0.03)	.83
	5.64 [5.62;5.67]	5.65 [5.63;5.67]	
**Total white matter lesion**	3.04 (0.60)	3.16 (0.63)	.96
	3.04 [2.54;3.43]	3.25 [2.62;3.56]	

Analysis of variance (ANCOVA) with total intracranial volume (TIV), type of scanner, age and gender as a covariate.
Abbreviations: mm³=millimeters cube, SD= standard deviation.

Figure e-1 Patients with LOD (n=36) and healthy controls (HC) (n=30) that had abnormal scores on the clinical scales when using cut-offs

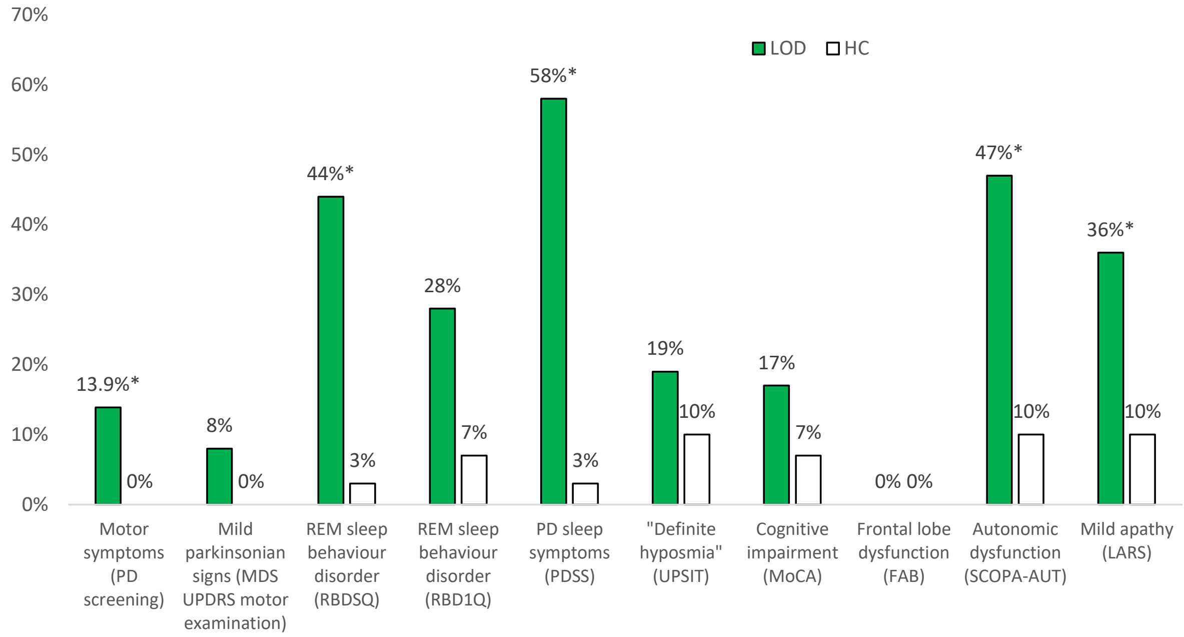

*p<0.05 using a Fisher's Exact between participant groups

LOD= late onset depressive disorder, PD screening= Parkinson's disease screening questionnaire, MPS= Mild Parkinsonian signs, RBD1Q= REM Sleep Behavior Disorder Single-Question Screen, RBDSQ= The REM sleep Behaviour Disorder screening questionnaire, PDSS=Parkinson's disease sleep scale, UPSIT= University of Pennsylvania Smell Identification Test, MoCA= Montreal Cognitive Assessment, FAB=Frontal Assessment Battery, SCOPA-AUT= Scales for Outcomes in Parkinson's disease- Autonomic, , LARS= Lille Apathy Rating Scale

References for the clinical assessments and cut offs used:

Kroenke K, Spitzer RL, Williams JB. The PHQ-9: validity of a brief depression severity measure. J Gen Intern Med. 2001;16(9):606-613.

Zigmond AS, Snaith RP. The hospital anxiety and depression scale. Acta psychiatrica Scandinavica. 1983;67(6):361-370.

Goetz CG, Tilley BC, Shaftman SR, et al. Movement Disorder Society-sponsored revision of the Unified Parkinson's Disease Rating Scale (MDS-UPDRS): scale presentation and clinimetric testing results. Movement disorders: official journal of the Movement Disorder Society. 2008;23(15):2129-2170.

Nasreddine ZS, Phillips NA, Bedirian V, et al. The Montreal Cognitive Assessment, MoCA: a brief screening tool for mild cognitive impairment. Journal of the American Geriatrics Society. 2005;53(4):695-699.

Dubois B, Slachevsky A, Litvan I, Pillon B. The FAB: a Frontal Assessment Battery at bedside. Neurology. 2000;55(11):1621-1626.

Sockeel P, Dujardin K, Devos D, Deneve C, Destee A, Defebvre L. The Lille apathy rating scale (LARS), a new instrument for detecting and quantifying apathy: validation in Parkinson's disease. Journal of neurology, neurosurgery, and psychiatry. 2006;77(5):579-584.

Postuma RB, Arnulf I, Hogl B, et al. A single-question screen for rapid eye movement sleep behavior disorder: a multicenter validation study. Movement disorders: official journal of the Movement Disorder Society. 2012;27(7):913-916.

Stiasny-Kolster K, Mayer G, Schafer S, Moller JC, Heinzel-Gutenbrunner M, Oertel WH. The REM sleep behavior disorder screening questionnaire--a new diagnostic instrument. Movement disorders: official journal of the Movement Disorder Society. 2007;22(16):2386-2393.

Chaudhuri KR, Pal S, DiMarco A, et al. The Parkinson's disease sleep scale: a new instrument for assessing sleep and nocturnal disability in Parkinson's disease. Journal of neurology, neurosurgery, and psychiatry. 2002;73(6):629-635.

Kim JH, Cheong HK, Lee CS, Yi SE, Park KW. The validity and reliability of a screening questionnaire for Parkinson's disease in a community. Journal of preventive medicine and public health = Yebang Uihakhoe chi. 2010;43(1):9-17.

Visser M, Marinus J, Stiggelbout AM, Van Hilten JJ. Assessment of autonomic dysfunction in Parkinson's disease: the SCOPA-AUT. Movement disorders: official journal of the Movement Disorder Society. 2004;19(11):1306-1312.

Doty RL, Shaman P, Kimmelman CP, Dann MS. University of Pennsylvania Smell Identification Test: a rapid quantitative olfactory function test for the clinic. The Laryngoscope. 1984;94(2 Pt 1):176-178.

For calculating the MPS, MoCA, hyposmia and axial symptoms, bradykinesia, rigidity and tremor cut offs;

Noyce AJ, Schrag A, Masters JM, Bestwick JP, Giovannoni G, Lees AJ. Subtle motor disturbances in PREDICT-PD participants. J Neurol Neurosurg Psychiatry. 2017;88(3):212-217.

Luis CA, Keegan AP, Mullan M. Cross validation of the Montreal Cognitive Assessment in community dwelling older adults residing in the Southeastern US. International journal of geriatric psychiatry. 2009;24(2):197-201.

Noyce AJ, Bestwick JP, Silveira-Moriyama L, et al. PREDICT-PD: identifying risk of Parkinson's disease in the community: methods and baseline results. J Neurol Neurosurg Psychiatry. 2014;85(1):31-37.

Li X, Xing Y, Martin-Bastida A, Piccini P, Auer DP. Patterns of grey matter loss associated with motor subscores in early Parkinson's disease. NeuroImage Clinical. 2018;17:498-504.
